# *Faecalibacterium duncaniae* A2‐165 growth is strongly promoted by yeast extract and vitamin B5 in cGMP medium

**DOI:** 10.1111/1751-7915.14374

**Published:** 2023-11-29

**Authors:** Lea Bircher, Alain M. Sourabié, Marijana Paurevic, Janina Hochuli, Annelies Geirnaert, Chloé Navas, Benoît Drogue, Christophe Lacroix

**Affiliations:** ^1^ Department Health Science and Technology, Laboratory of Food Biotechnology, Institute of Food, Nutrition and Health ETH Zurich Zürich Switzerland; ^2^ Science Technology and Innovation Department Procelys by LeSaffre Maisons‐Alfort France

## Abstract

Several gut microbial species within the *Faecalibacterium* genus have emerged as promising next‐generation probiotics (NGP) due to their multifunctional protective effects against gastrointestinal and systemic disorders. To enable clinical studies and further applications, improved methods for cultivating *Faecalibacterium* must be developed in compliance with current Good Manufacturing Practice regulations, which is complicated by its oxygen sensitivity and complex nutritional requirements. Different yeast‐based nutrients (YBNs), including yeast extracts (YEs) and yeast peptones (YPs), are ubiquitously used when cultivating microbes to supply a broad range of macro‐ and micronutrients. In this study, we evaluated six experimental YBNs, namely three YEs, two YPs and a yeast cell wall product (YCW), and eight B‐vitamins in the cultivation of *Faecalibacterium duncaniae* A2‐165, former *Faecalibacterium prausnitzii*, using growth assays in microtitre plates, dose‐effect studies in Hungate tube fermentations and fully controlled bioreactor experiments. We demonstrated that YEs promote *F. duncaniae* A2‐165 growth in a nutritionally limited medium, while YPs and YCW lacked essential growth factors for enabling cell propagation. High cell density was obtained in controlled bioreactors using a medium containing 2–4% of a selected YE and 1% casein peptone (3.4 ± 1.7 × 10^9^–5.1 ± 1.3 × 10^9^ cells mL^−1^). Among all tested B‐vitamins, we identified B5 as a strong growth promoter. Replacing casein peptone with YP and supplementing with vitamin B5 further increased biomass by approximately 50% (6.8 ± 1.7 × 10^9^ cells mL^−1^). Hence, empirical selection of YE, YP and B5 allowed formulation of a high‐yielding animal allergen‐free nutritive medium to produce *F. duncaniae* A2‐165. Selecting nutritionally suitable YBNs and combining these with other key nutrients are important steps for optimizing production of NGP with high yields and lower cost.

## Graphical abstract

In this study, an animal allergen‐free production medium for the next‐generation probiotic *Faecalibacterium duncaniae* A2‐165 was designed and evaluated using a stepwise approach. Different yeast‐based nutrients and B‐vitamins were tested, allowing the design of an effective medium formulation containing yeast extract, yeast peptone and vitamin B5 that yielded high *F. duncaniae* A2‐165 cell numbers (6.8 ± 1.7 × 10^9^ cells mL^−1^) in bioreactor experiments. Selecting nutritionally suitable yeast‐based nutrients and combining these with other key nutrients are important steps for optimizing production of next‐generation probiotics with high yields and lower cost.

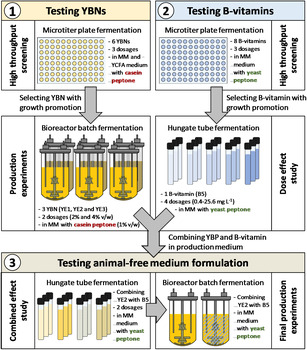

## INTRODUCTION

The genus *Faecalibacterium* encompasses several species that are highly prevalent members of the human gut microbiota and are key for host health (Miquel et al., 2013; Sakamoto et al., 2022; Zou et al., 2021). *Faecalibacterium* abundance was shown to be negatively correlated with distinct diseases, including different types of cancers, atopic disease and intestinal and metabolic disorders (Björkqvist et al., 2019; Ma et al., 2020; Song et al., 2016; Touch et al., 2022; Touchefeu et al., 2021). Consequently, several *Faecalibacterium* species are currently being explored as next‐generation probiotics (NGPs) with promising results in murine models of inflammation, colitis, diabetes and obesity, which is partly associated with the production of butyrate (De Filippis et al., 2022; Miquel et al., 2013) and Microbial Anti‐inflammatory Molecule, a key element of its anti‐inflammatory activity (Auger et al., 2022). Butyrate serves as both an energy source for colonocytes and an anti‐inflammation promotor within the intestinal tract (Lenoir et al., 2020; Salvi & Cowles, 2021). However, to ensure successful translation of this NGP, a robust and high‐yield cultivation process must be developed, which would allow for clinical studies and ultimately, market distribution. However, cultivating *Faecalibacterium* spp. is still very challenging due to its extreme oxygen sensitivity and mostly unknown nutritional requirements (Andrade et al., 2020). For instance, members of the genus are considered auxotrophic for various B‐vitamins, as they lack the genes involved in biosynthesis of several amino acids (Rodionov et al., 2019; Soto‐Martin et al., 2020). Besides considering nutritional aspects, a culture medium compliant with current Good Manufacturing Practice (cGMP) and free of animal and allergenic ingredients must be developed for *Faecalibacterium* spp. (Cordaillat‐Simmons et al., 2020). Yeast‐based nutrients (YBNs), namely yeast extracts (YEs) and yeast peptones (YPs), are complex nutrient sources of non‐animal origin that contain a variety of essential growth factors like peptides of various sizes, free amino acids, vitamins, proteins, amino acids, minerals, nucleic bases and trace elements (Doo et al., 2017; Grant & Pramer, 1962). Besides, reported cases of yeast allergies are extremely rare compared to the prevalence of allergies towards animal‐derived proteins (Pajno et al., 2005). YBNs are commonly added to cultivation media at defined levels in concentrations ranging from 0.1% to 4% to produce bacterial biomass (Fenster et al., 2019). However, the composition of different YBNs can be very diverse, and is impacted by yeast type and up‐ and downstream processing (Proust et al., 2019). Therefore, it is crucial to carefully select a YBNs or a mix thereof that contain the optimal combination of nutrients and growth factors to meet the nutritional needs of the target bacterial strain. Absent or reduced amounts of nutritional factors in the YBN can greatly affect growth performance, leading to lower cell yields (Fenster et al., 2019; Nancib et al., 1991). To overcome potential nutritional limitations of YBN, essential strain‐specific growth‐promoting factors need to be identified and added to the production medium to ensure optimal growth.

In this work, we employed a stepwise approach to developing a cGMP‐compliant medium free of animal derived ingredients to produce *Faecalibacterium duncaniae* A2‐165 (previously *Faecalibacterium prausnitzii* A2‐165) at high cell yields. The selected strain is the most extensively studied and most promising representative among the various *Faecalibacterium* spp. being considered as NGPs, with demonstrated therapeutic efficiency in murine models of asthma, colitis and chronic inflammation (De Filippis et al., 2022). *Faecalibacterium duncaniae* A2‐165 produces the short‐chain fatty acid butyrate as a major end‐product of glucose fermentation under net‐acetate consumption and forms minor portions of lactate and formate (Duncan, Hold, et al., 2002; Sakamoto et al., 2022). Furthermore, unlike other *Faecalibacterium* spp., this strain exhibits limited production of exopolysaccharides which can result in biofilm formation that hinders downstream processing (Rossi et al., 2015). Initially, we evaluated the nutritional and growth‐promoting properties of six experimental YBNs, corresponding to different yeast fractions of *Saccharomyces cerevisiae* or production processes, to cultivate *F. duncaniae* A2‐165. Secondly, we screened for essential nutritional compounds with growth promoting effects, focusing on B‐vitamins, given their demonstrated importance for the growth of *Faecalibacterium* spp. (Soto‐Martin et al., 2020). We then assessed dose effects under different YBNs and B‐vitamin concentrations in Hungate tube fermentations. Finally, we formulated a production medium using YBN alone or supplemented with vitamin B5 to optimize cell yield and conducted *F. duncaniae* A2‐165 batch fermentation trials in fully controlled bioreactors.

## EXPERIMENTAL PROCEDURE

### Experimental set‐up

**FIGURE 1:**
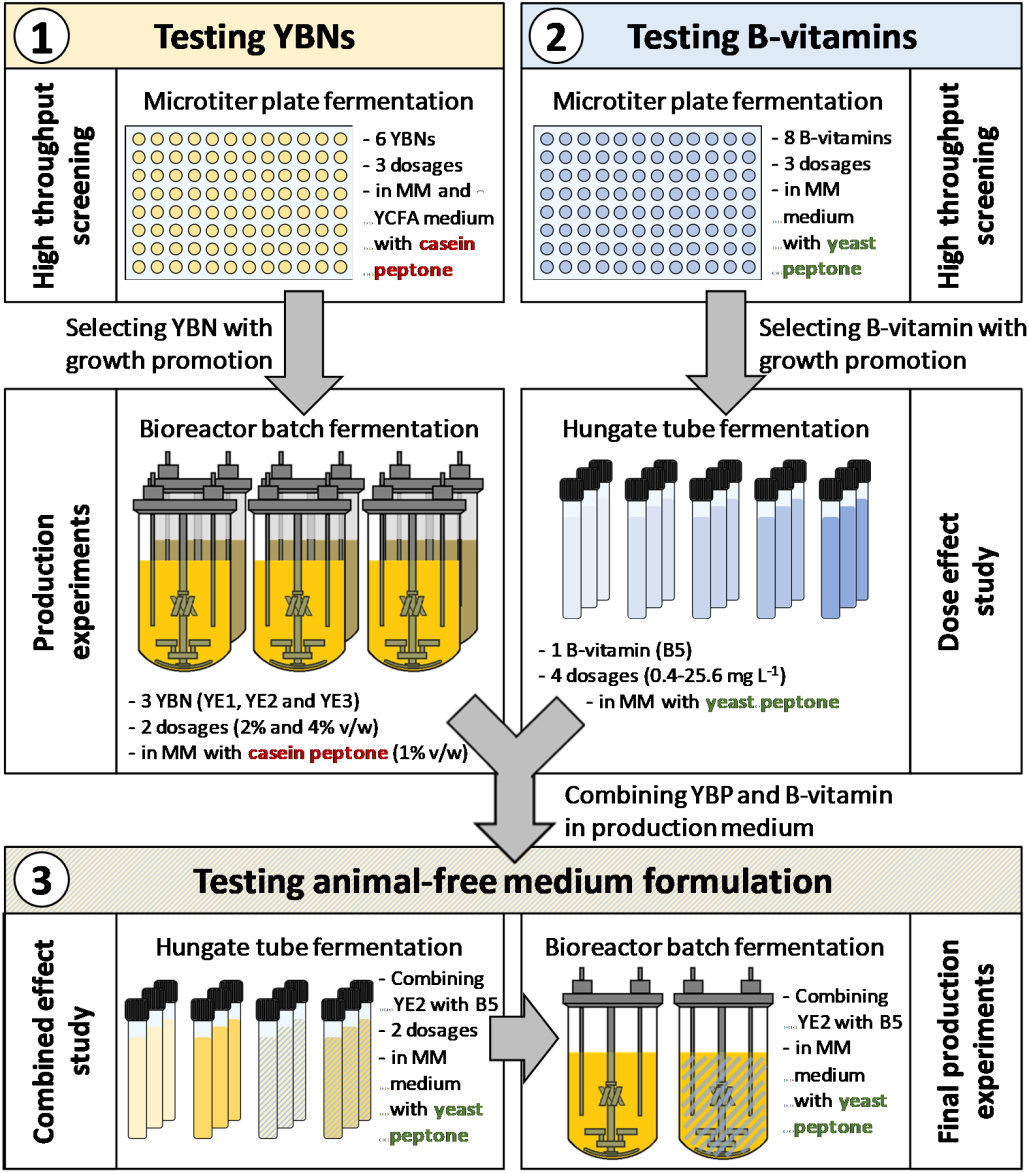
Experimental set‐up. The study consisted of three parts. The first part investigated the nutritional and production potential of six different yeast‐based nutrients (YBNs) on *F. duncaniae* A2‐165 in minimal medium (MM) and yeast‐casitone‐fatty‐acids medium (YCFA) containing casein peptone. The growth‐promoting effect of eight different B‐vitamins was tested in the second part and in the third part, *F. duncaniae* production tests were conducted to investigate the most effective combination of YBN and B‐vitamin in medium formulation. Casein peptone was replaced by yeast peptone in parts two and three to obtain a fully animal‐free medium composition.

The study applied a stepwise approach, built on three main parts (Figure 1), with an ultimate objective of formulating a cGMP‐compliant medium free of animal‐derived ingredients enabling the production of high cell yields of *F. duncaniae* A2‐165. The first part involved screening six different YBNs by first testing the growth of *F. duncaniae* in microtitre plates under different YBN conditions followed by production tests in bioreactors, using the three YBNs that demonstrated the best growth performance in the screening experiments. The medium used in this first part contained casein peptone, which was replaced by yeast peptone in the subsequent parts because of the animal origin and potential allergenicity of casein peptone. In the second part, the growth‐promoting effect of eight B‐vitamins at different dosages was tested on *F. duncaniae* growth in microtitre plates. Hungate tube fermentations were then conducted to test the dose–response of vitamin B5, which had demonstrated growth‐promoting effects in earlier microtitre plate tests. In the final part, information from the previous two parts was used to design and investigate a production medium that contained an effective YBN (YE2) and B‐vitamin (B5) combination. The additive effect of B5 addition at two different dosages to YE2 was first investigated in Hungate tube fermentation and the most efficient combination was then tested in controlled bioreactor experiments.

### Bacterial strain and inoculum preparation

*Faecalibacterium duncaniae* A2‐165, formerly classified as *Faecalibacterium prausnitzii* A2‐165 (Duncan, Hold, et al., 2002; Sakamoto et al., 2022), was obtained from the Deutsche Sammlung für Mikroorganismen und Zellkulturen (DSMZ, Braunschweig, Germany). *Faecalibacterium duncaniae* A2‐165 stock cultures were prepared and maintained in a 15% glycerol suspension (Sigma‐Aldrich, Buchs, Switzerland) at −80°C as described previously (Bircher et al., 2018). To produce the inoculum, 10 mL of YCFA (yeast extract, casitone and fatty acid) medium in a Hungate tube was inoculated anaerobically with 0.5 mL of a reactivated stock culture. The inoculum was subcultivated twice at 2% (v/v) inoculation rate and incubated anaerobically for 12 h at 37°C to produce a working culture. YCFA medium was prepared as described previously (Duncan, Barcenilla, et al., 2002), with two adaptations: glucose (6 g L^−1^, Sigma‐Aldrich, Buchs, Switzerland) was added as sole carbon source and all components of the volatile fatty acid mix were omitted except acetate (33 mM, Sigma‐Aldrich, Buchs, Switzerland), which *Faecalibacterium* spp. can use as a carbon source (Khan et al., 2012). The YE component of the YCFA medium consisted of a particular yeast extract for microbiology (2.5 g L^−1^, Sigma‐Aldrich, Buchs, Switzerland) that was not included in the selection of the test YBNs.

### Preparation of growth media with different YBNs

To evaluate the nutritional effect of different YBNs, a minimal medium (MM) was designed that only contained essential minerals, a small amount of casein peptone (2 g L^−1^, Sigma‐Aldrich, Buchs, Switzerland) and glucose as growth substrate (Table S1). A nutritionally richer YCFA‐like medium (Table S2) lacking the YE component was used to evaluate the cell production potential of the different YBNs. Compared to MM, YCFA medium was supplemented with essential vitamins, bovine hemin as iron source (Sigma‐Aldrich, Buchs, Switzerland) in place of iron sulphate in MM, and 10 g L^−1^ casein peptone as a source of peptides and amino acids. Three YEs (YE1, YE2 and YE3), two YPs (YP1 and YP2) and one yeast cell wall product (YCW) were selected for this study (Procelys by Lesaffre, Maisons‐Alfort, France), all deriving from *Saccharomyces cerevisiae* but differing in their nucleotide, B‐vitamin and iron contents. All selected YBNs are still in experimental stage, and not yet available on the commercial market. The main characteristics of the six YBNs are presented in Table S3. The different YBNs were weighed in 15‐mL Falcon tubes (0.45 g) and stored overnight in an anaerobic chamber (10% CO_2_, 5% H_2_ and 85% N_2_; Coy Laboratories, MI, USA) to remove traces of oxygen. Thereafter, YBNs were dissolved in 10 mL of either MM or YCFA medium and filter‐sterilized into a sterile Falcon tube.

### Assessment of *F. duncaniae* growth performance under different YBNs conditions

Growth was evaluated under anaerobic conditions in 96‐well microtitre plates in quadruplicate, as described previously (Bircher et al., 2018). Inner wells of a microtitre plate, reduced overnight in an anaerobic chamber, were filled with 180 μL of a mixture containing YCFA or MM medium, a glucose solution (final concentration of 5.00 g L^−1^) and YBN solution added at final concentrations of 1.25, 2.50 or 5.00 g L^−1^. Wells were inoculated with 20 μL (10% v/v) of fresh *F. duncaniae* inoculum. Wells in outer rows and columns were filled with activated carbon of an AnaeroGen bag (Thermo Fisher Diagnostics AG, Pratteln, Switzerland). Plates were covered with a ClearSeal film (Labgene Scientific Instruments, Châtel‐Saint‐Denis, Switzerland) and lid, which was sealed with petroleum jelly to maintain anoxic conditions during incubation outside of the anaerobic chamber. Growth was monitored at 37°C by recording optical density (OD) at 600 nm every 15 min in a microplate reader (Powerwave XS, Winooski, USA). To analyse the growth kinetics of *F. duncaniae*, OD600 values were fitted using the ‘growth rates made easy method’ by Hall et al. (2014). The maximal increase in optical density, termed as carrying capacity (ΔOD) and the maximum growth rate (μ_max_) were calculated by fitting segments of linear models to the log‐transformed data during exponential growth using the package ‘growthrates’ in the statistics program RStudio 1.3.1093 (Boston, MA, USA).

### Preparation of growth media with different B‐vitamins

The growth‐promoting potential of eight B‐vitamins (Sigma‐Aldrich, Buchs, Switzerland); namely, thiamine (B1), riboflavin (B2), niacin (B3), pantothenate (B5), pyridoxine (B6), biotin (B7), folate (B9) and cobalamin (B12), was evaluated in MM medium (Table S1) that was slightly modified by replacing casein peptone with yeast peptone (YP2) and increasing the concentration to 10 g L^−1^ to allow growth under low nutrient conditions. Removing casein peptone and replacing hemin by iron sulphate allows progress towards a medium free of animal‐derived potential allergenic ingredients, which is a crucial requirement for biotherapeutic production. The MM was anaerobically prepared, and the B‐vitamins were added as single components or combined, at three different concentrations (Table S4). The lowest concentrations of B‐vitamins added were based on levels found in the human gut (Michel et al., 1998), and were duplicated and quadruplicated to obtain medium and high concentrations, respectively.

### Assessment of *F. duncaniae* growth performance under different B‐vitamin conditions

#### High‐throughput screening

The B‐vitamin screening experiments were carried out in quadruplicate growth assays in 96‐well microtitre plates (Bioswisstec AG, Schaffhausen, Switzerland) using a plate reader (Tecan Infinite® 200 PRO, Männedorf, Switzerland) in an anaerobic chamber. Wells were filled with 180 μL medium with different B‐vitamin treatments and inoculated with 20 μL of an overnight culture of *F. duncaniae* (10% v/v). Microtiter plates were incubated at 37°C for 24 h and the OD was measured at 600 nm at intervals of 30 min. The plates were covered with a Breath‐Easy sealing membrane (Sigma‐Aldrich, Buchs, Switzerland) to allow gas exchange while preventing excessive evaporation. Before each OD measurement, the plates were shaken for 5 s with an amplitude of 2 mm followed by a waiting time of 10 s. ΔOD and μ_max_ were calculated as described for the microtitre plate growth assay with different YBN.

#### Hungate tube fermentation

Hungate tube fermentations were conducted in quadruplicate growth experiments to determine the optimal B5 concentration for *F. duncaniae* growth for final production medium formulation. Therefore, Hungate tubes containing 10 mL MM were supplemented with different B5 doses (0.4, 1.6, 6.4 and 25.6 mg L^−1^), inoculated with an overnight culture of *F. duncaniae* at 2% (v/v) and incubated at 37°C for 24 h. The OD was measured with a cell density photometer (Labgene Scientific, Châtel‐Saint‐Denis, Switzerland) at 600 nm after 0, 2, 4, 6, 8, 11 and 24 h and compared to a control without added B5.

### Production trials in bioreactors

The *F. duncaniae* production trials were carried out in glass bioreactors (Multifors, Infors, Bottmingen, Switzerland or DASbox®, Vaudaux‐Eppendorf AG, Basel, Switzerland) operated for 28 h in batch mode with a volume of 200 mL. The strain was cultivated in a MM medium (Duncan et al., 2003) containing 10 g L^−1^ peptone, 25 g L^−1^ glucose and 66 mM acetic acid (Table S5). The pH of the medium was initially set to 6.5 and controlled at 6.0 by adding NaOH (2.5 N). Temperature was maintained at 37°C, strict anaerobiosis was upheld by continuous flow of CO_2_ into the headspace of the reactors (0.1 L min^−1^), and stirring was set to 120 rpm. The reactors were inoculated at 1% (v/v) with a fresh overnight *F. duncaniae* culture, aiming for an initial concentration of 10^6^ cells mL^−1^. Temperature, pH, redox potential and base consumption were monitored throughout the experiment. For metabolite analysis and cell enumeration, 1‐mL samples were taken at different time points and centrifuged at 14000×*g* for 5 min at 4°C. Supernatant samples stored at −20°C were used for HPLC‐IR analysis and microbial pellets stored at −80°C for DNA extraction. The first production trial aimed to investigate the effect of the three different YEs (YE1, YE2 and YE3), supplemented at concentrations of 20 or 40 g L^−1^ (low and high dose), on biomass production and metabolism of *F. duncaniae*. Therefore, six test reactors were set up, each containing one of the three YE at low (2% v/w) or high dose (4% v/w), and independent triplicate fermentations were conducted. The second production trial evaluated the growth‐promoting effect of B5 addition (54.2 mg L^−1^) to the cultivation medium containing 40 g L^−1^ YE2 and compared to a control without B5. Batch experiments were run in independent triplicates with a two‐reactor configuration and under the same conditions as described for the first production trial, except casein peptone was replaced by yeast peptone (YP2) and the operation time was 30 h.

### Measurement of metabolites

HPLC analyses were performed to quantify the concentrations of glucose, acetate, butyrate and the intermediate metabolites lactate and formate. Analyses were conducted using an Accela chromatography system (Thermo Fisher Scientific, MA, USA) equipped with a Carbon‐H cartridge (4 × 3.0 mm) connected to a Rezex® ROA‐Organic Acid (300 × 7.8 mm) column (Phenomenex Helvetia GmbH, Basel, Switzerland) and an Accela refraction index detector (Thermo Fisher Scientific, MA, USA). Supernatants were filtered through a 0.45 μm nylon membrane (Infochroma AG, Zug, Switzerland) into glass vials and sealed with crimp caps. Filtered supernatants (20 μL injection volume) were eluted with 10 mM H_2_SO_4_ at a flow rate of 0.4 mL min^−1^ at 40°C. Sugars, short‐chain fatty acids and intermediate metabolites were quantified using external standards (Sigma‐Aldrich, Buchs, Switzerland).

### Quantitative real‐time polymerase chain reaction for enumeration of cell concentration

Total genomic DNA of the cell pellets was extracted using the MP Kit for soil kit (MP Biomedicals, France) according to the manufacturer's instructions. Quantitative real‐time polymerase chain reaction (qPCR) targeting the *F. prausnitzii*‐specific 16S rRNA gene regions using the primers Fprau223F and Fprau420R (Bartosch et al., 2004) was used for cell quantification. The samples were run in duplicate in 96‐well plates (Roche Diagnostics GmbH) in a total reaction volume of 10 μL containing 1 μL DNA template (1:10 or 1:100 diluted with MilliQ water). The composition of the reaction mix for one sample is listed in Table S6. The 96‐well plate was sealed with an optically clear film (Roche Diagnostics GmbH, Rotkreuz, Switzerland) and the qPCR was performed by the Roche LightCycler® 480 II (Roche Diagnostics GmbH, Rotkreuz, Switzerland). The amplification started with an initial denaturation step at 95°C for 10 min, followed by 40 amplification cycles at 95°C for 15 s and 60°C for 1 min. Data analysis was conducted with the LightCycler® 480 software version 1.5.1.62. Melting curve analysis was performed to verify the specificity of amplification. Standard curves were generated from 10‐fold dilution series (10^2^–10^8^ copies) of linearized plasmids containing the target 16S rRNA genes and were used to calculate the gene copy number per μL reaction mix, expressed per mL effluent. To obtain the absolute cell concentration per mL effluent, gene copy numbers were divided by six to account for the number of 16S rRNA copies present in the *F. duncaniae* genome (Stoddard et al., 2015).

### Statistics

Statistical analysis was done using R studio version 1.3.1056 (Boston, MA, USA). A two–way ANOVA with interaction term followed by Tukey's HSD (honestly significant difference) was performed to test for significant effect of the factors YBN or dosage on *F. duncaniae* growth performance (ΔOD and μ_max_). The model was selected based on the Akaike information criterion explaining the largest amount of variation in the response variable. Differences were considered significant for *α* ≤ 0.05. In addition, an unpaired, two‐tailed *t*‐test was performed to determine significant differences between the three dosages on ΔOD and μ_max_ within the same YBN treatment and between media (YCFA and MM). A two–way ANOVA with interaction term was also used to test the significance of the effects of B‐vitamin and dosage on growth performance in the microtitre plate growth assay. An interaction term was omitted for testing YBN and dosage on cell yield and metabolite production in the first production trial in bioreactors, as it did not improve model fit. For the results of the dose‐effect study in Hungate fermentation and the production tests with B5 supplementation, an unpaired, two‐tailed *t*‐test was performed to detect significant effects on cell yield and metabolite production between the different B5 dosages and two growth media, respectively. Differences were considered significant for *α* ≤ 0.05.

## RESULTS

### Yeast extracts promote *F. duncaniae* A2‐165 growth in a product‐ and dose‐dependent manner

**FIGURE 2:**
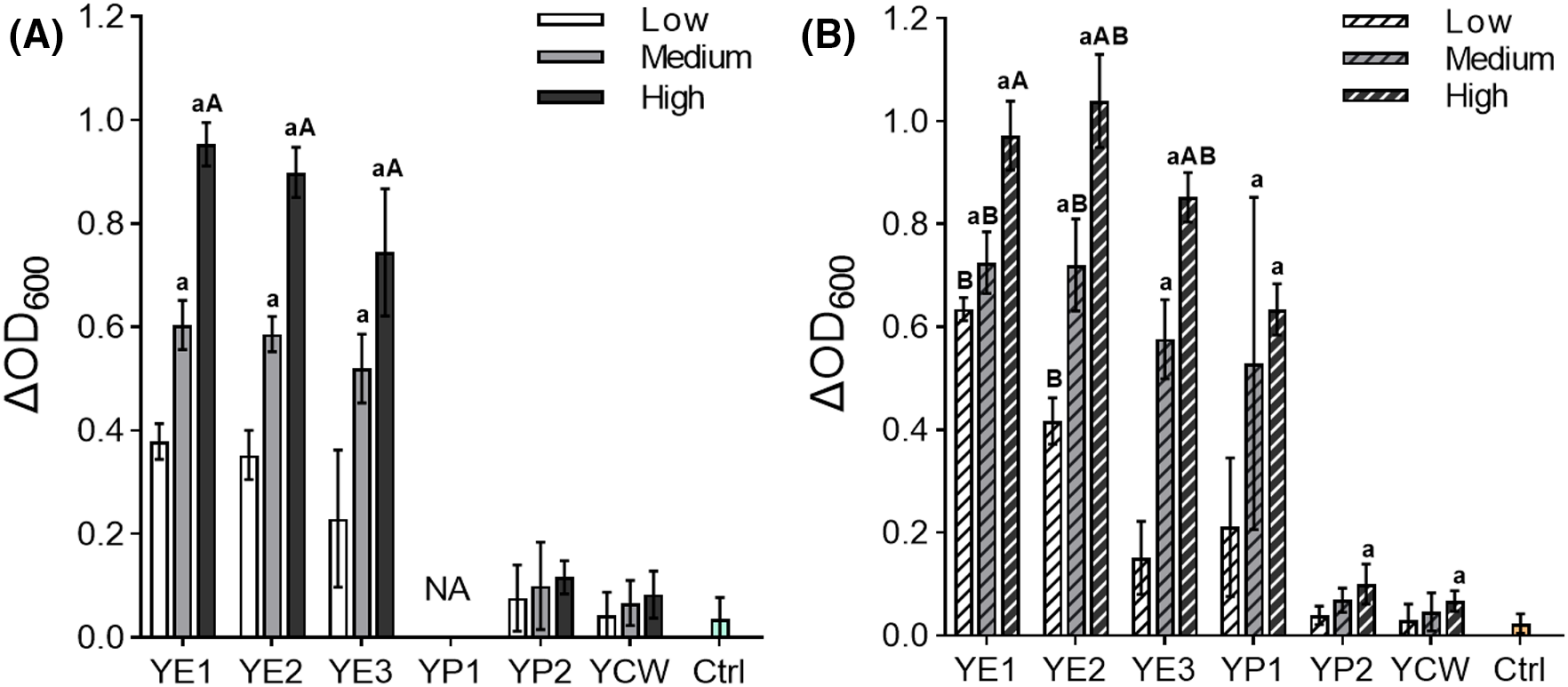
Impact of six different yeast‐based nutrients (YBNs) in different dosages on carrying capacity (ΔOD) of *F. duncaniae* A2‐165 in microtiter plates. Bars represent the mean values of max. ΔOD of quadruplicate growth experiments using minimal (A) or yeast‐casitone‐fatty‐acids medium (B) supplemented with low (1.25 g L^−1^), medium (2.50 g L^−1^) or high (5.00 g L^−1^) YBN concentrations or a control without YBN (Ctrl). No growth was detected in minimal medium under YP1 condition (NA). Statistics were performed by unpaired *t*‐test, comparing for each YBN the ΔOD values of different dosage and medium. Bars marked with an a are significantly different from the low dosage and bars marked with an A from the medium dosage within the same medium and YBN treatment, while bars marked with B are significantly different between the same dosage and YBN treatment of different media.

In the first step, the nutritional potential of several YBNs (three yeast extracts: YE1, YE2, YE3, two yeast peptones: YP1 and YP2 and an enzymatically digested yeast cell wall product: YCW) at low (1.25 g L^−1^), medium (2.50 g L^−1^) and high dosages (5.00 g L^−1^) were evaluated on the growth performance (μ_max_ and ΔOD) of *F. duncaniae* A2‐165 during anaerobic incubation in 96‐well microtitre plates. YBNs were added to a MM consisting of essential minerals, a small amount of casein peptone as nitrogen source (2.00 g L^−1^) and glucose as growth substrate (5.00 g L^−1^). Among all tested YBNs and dosages, only the YEs showed a growth‐promoting effect on *F. duncaniae* A2‐165 (Figure 2A). Adding YE1, YE2 and YE3 to MM resulted in a significantly higher growth (ΔOD measured at 600 nm) than in the control without YBNs (*p* < 0.001; Table S7). Comparable growth was obtained with YE1 (0.45 ± 0.24) and YE2 (0.42 ± 0.23), with both producing significantly higher growth than with YE3 (0.31 ± 0.25, *p* < 0.001; Table S7). The supplemented YE concentration greatly influenced growth (*p* < 0.001; Table S8). This effect was most pronounced with YE3, depicted by a ΔOD of 0.23 ± 0.16, 0.52 ± 0.07 and 0.74 ± 0.12 with low, medium and high YE concentrations, respectively, which corresponded to a 3.2‐fold increase between low and high dose. High doses of YE1 and YE2 in MM resulted in similar ΔOD of 0.95 ± 0.04 and 0.90 ± 0.05, respectively, which were 2.5‐fold higher compared to the low dose (0.38 ± 0.03 and 0.35 ± 0.05) and significantly higher than with high YE3 treatment (*p* < 0.001 and *p* < 0.005). In contrast, the dosage factor had no effect on *F. duncaniae* A2‐165 growth rate μ_max_ (*p* = 0.825; Table S8). The highest values of μ_max_ were measured with YE2 supplementation (0.83 ± 0.07–0.86 ± 0.07 ΔOD h^−1^) and were two‐fold higher than for YE1 and YE3 treatment (Table 1). Altogether, these observations indicate that YE1 and YE2 offer the highest nutritional potential for *F. duncaniae* A2‐165 cell production among the tested YBNs.

**TABLE 1:** Growth rate (μ_max_) of *Faecalibacterium duncaniae* A2‐165 in MM and YCFA‐like medium supplemented with YE1, YE2, YE3 and YP1 at different dosages in microtitre plates.

YBN	μ_max_ in MM [ΔOD h^−1^]			μ_max_ in YCFA [ΔOD h^−1^]		
	Low [1.25 g L^−1^]	Medium [2.50 g L^−1^]	High [5.00 g L^−1^]	Low [1.25 g L^−1^]	Medium [2.50 g L^−1^]	High [5.00 g L^−1^]
YE1	0.42 ± 0.14	0.43 ± 0.09	0.59 ± 0.11^a^, ^A^	0.44 ± 0.06^B^	0.47 ± 0.04	0.53 ± 0.07
YE2	0.86 ± 0.07	0.86 ± 0.05	0.83 ± 0.07	0.73 ± 0.07^B^	0.78 ± 0.05^B^	0.90 ± 0.10^a^, ^A^
YE3	0.46 ± 0.15	0.46 ± 0.08	0.47 ± 0.10	0.42 ± 0.03	0.45 ± 0.10	0.53 ± 0.01^a^, ^A^, ^B^
YP1	No growth detected			0.29 ± 0.06	0.26 ± 0.08	0.47 ± 0.07^a^, ^A^

Abbreviations: YBN, yeast‐based nutrient; YCFA, yeast‐casitone‐fatty‐acids; YE, yeast extract.

^a^ Indicates that μ_max_ of the medium or high dosage is significantly different from the low dosage within the same medium and YBN.

^A^ Indicates that μ_max_ of the high dosage is significantly different from the medium dosage within the same medium and YBN.

^B^ Indicates that μ_max_ of an YBN is significantly different between different media.

In the second step, production yield of *F. duncaniae* A2‐165 with the different YBNs was tested, using a YCFA‐based medium that provided better nutritive conditions than MM by adding essential vitamins, bovine hemin as iron source and a five‐fold increased amount of casein peptone (10.0 g L^−1^). The YCFA‐based medium had a positive effect on growth of *F. duncaniae* A2‐165, resulting in significantly higher cell growth compared to MM over all tested conditions (*p* < 0.001). The dose‐dependent responses of the supplemented YEs observed in MM were kept in YCFA‐based medium (Figure 2B). Low YE1 condition resulted in a ΔOD of 0.64 ± 0.02, which was 1.7‐fold higher than that in MM (*p* < 0.001). In contrast, comparable growth in MM and YCFA medium was measured with YE1 supplemented at high dose (0.97 ± 0.07). The most pronounced dose‐dependent growth promotion in YCFA medium was observed with YE2. Both ΔOD and μ_max_ increased significantly with high‐ compared to low‐YE2 conditions, from a ΔOD of 0.42 ± 0.05 to 1.04 ± 0.09 (*p* < 0.001), and from 0.73 ± 0.07 ΔOD h^−1^ to 0.90 ± 0.10 ΔOD h^−1^ (*p* = 0.001). YE3 supplementation, especially at a high dose, resulted in significantly higher μ_max_ (0.58 ± 0.08 ΔOD h^−1^, *p* = 0.026) and ΔOD values (0.85 ± 0.05, *p* = 0.036) in YCFA than in MM. YCFA medium also enabled growth of *F. duncaniae* in the presence of YP1, albeit at lower levels than with the tested YEs. In contrast, neither YP2 nor YCW resulted in substantial growth under YCFA conditions (ΔOD <0.1). Based on the obtained cell yields, YE2 had the highest production potential of all tested YBNs.

### *Faecalibacterium duncaniae* A2‐165 bioreactor fermentations using different yeast extracts

**FIGURE 3:**
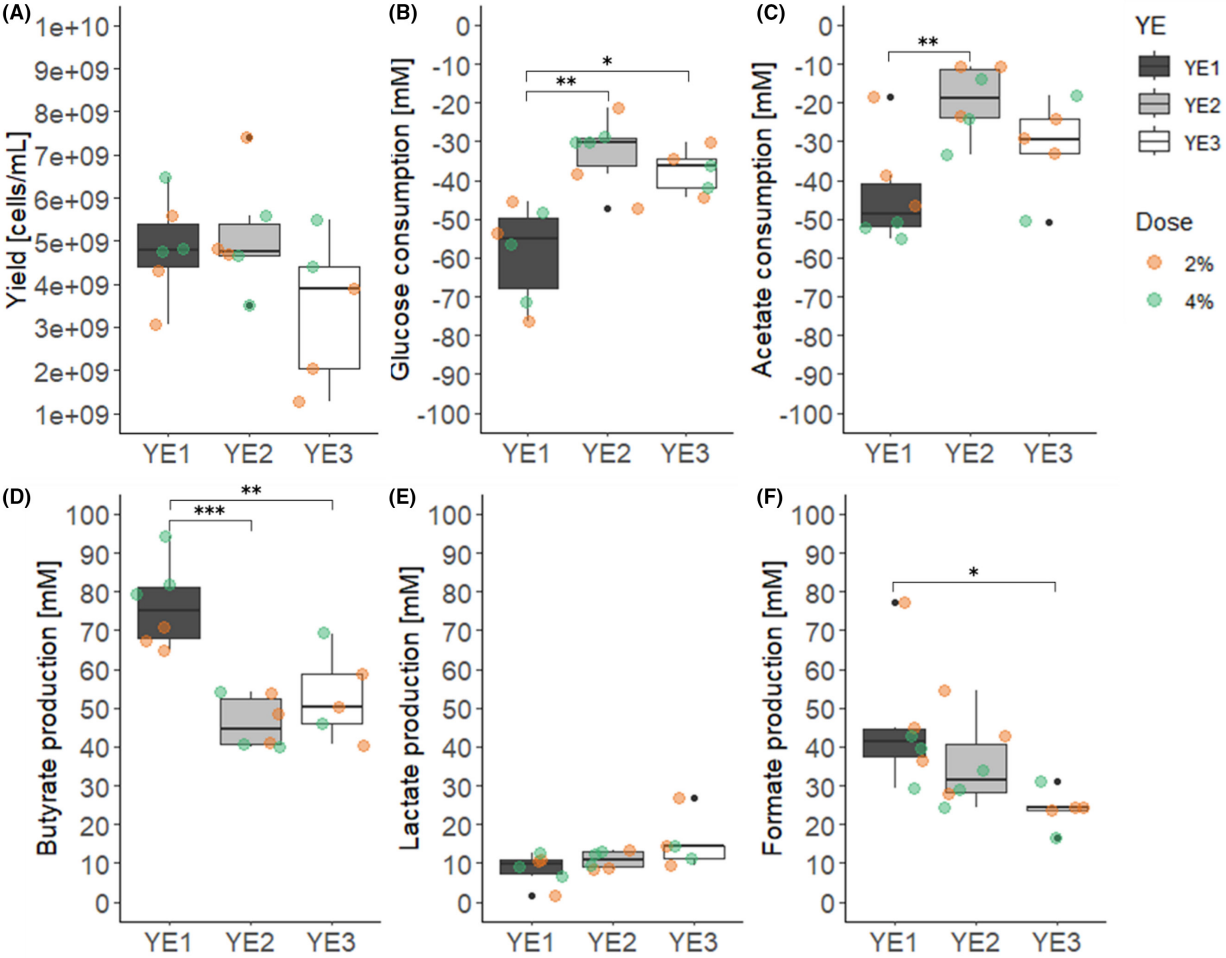
*F. duncaniae* A2‐165 cell yield, substrate consumption and metabolite production after 28 h batch fermentations in bioreactors using three different yeast extract (YE) types at two dosages. *F. duncaniae* A2‐165 was cultivated in yeast‐casitone‐fatty‐acids‐like medium containing 20 g L^−1^ () or 40 g L^−1^ YE () and 10 g L^−1^ casein peptone as nitrogen source. Cell yield and metabolite concentrations were measured in the spent media using quantitative real‐time polymerase chain reaction (qPCR) targeting the *F. duncaniae* A2‐165‐specific region of the 16S rRNA gene and HPLC‐RI, respectively. To obtain the cell number, qPCR values were divided by 6, corresponding to the number of 16S rRNA gene copies per cell. Boxes represent the first quartile, median and third quartile values of the highest measured cell yield (A) and end metabolite concentration (B) of independent triplicated growth experiments. Statistics were performed by two‐way ANOVA following a post‐hoc test using Tukey HST, comparing the means of all YE types, *p < 0.05, **p < 0.01, ***p < 0.001.

The stimulation effect of three selected YEs (YE1, YE2 and YE3) at low (20 g L^−1^) and high dosages (40 g L^−1^) on *F. duncaniae A2‐165* growth was tested during pH‐controlled batch fermentations in bioreactors using MM containing 10 g L^−1^ casein peptone, 25 g L^−1^ glucose (138 mM) and 66 mM acetic acid. Cell concentration, substrate consumption and metabolite production were monitored over 28 h batch fermentations. Substrate utilization and metabolite production significantly differed between the YEs. The metabolic activity was highest with *F. duncaniae* A2‐165 cultures grown on YE1, and lowest when grown on YE2 (Figure 3B–F). The addition of YE1 to the fermentation medium resulted in the consumption of 58.7 ± 12.5 mM glucose and 43.7 ± 13.6 mM acetate, leading to the formation of 76.4 ± 11.0 mM butyrate, 45.1 ± 16.6 mM formate and 8.5 mM lactate. *F. duncaniae* A2‐165 grown on YE2 only consumed half the glucose (32.7 ± 9.0 mM, *p* = 0.002) and acetate (19.4 ± 6.7 mM, *p* = 0.004), with a 40% decrease in butyrate formation (46.3 ± 6.7 mM, *p* < 0.001) compared to YE1. Similarly, *F. duncaniae* A2‐165 grown on YE3 produced 30% less butyrate (53.0 ± 11.3 mM, *p* = 0.003) and 50% less formate (24.1 ± 5.1 mM, *p* = 0.024) while consuming 60% less glucose (37.5 ± 5.8 mM, *p* = 0.012) than with YE1. Including an interaction term between YE type and dosage did not improve the ANOVA model fit, indicating that it had no significant effect on metabolic activity, while the factor dosage alone significantly impacted acetate levels (*p* = 0.009; Table S9) resulting in 11.1 mM higher consumption when doubling the YE dose. The maximal cell numbers among YEs showed no correlation with the varying metabolic yields observed between the YEs. Neither the type of YE nor the dose had a significant effect on cell numbers (Table S10). YE1, YE2 and YE3 addition resulted in comparable cell yields of 4.8 ± 1.2 × 10^9^, 5.1 ± 1.3 × 10^9^ and 3.4 ± 1.7 × 10^9^ cells mL^−1^ (Figure 3A), respectively, measured after 23.3 ± 2.7, 24.3 ± 2.9 and 23.6 ± 2.6 h incubation.

### Impact of essential B‐vitamins on *F. duncaniae* A2‐165 growth performance

**FIGURE 4:**
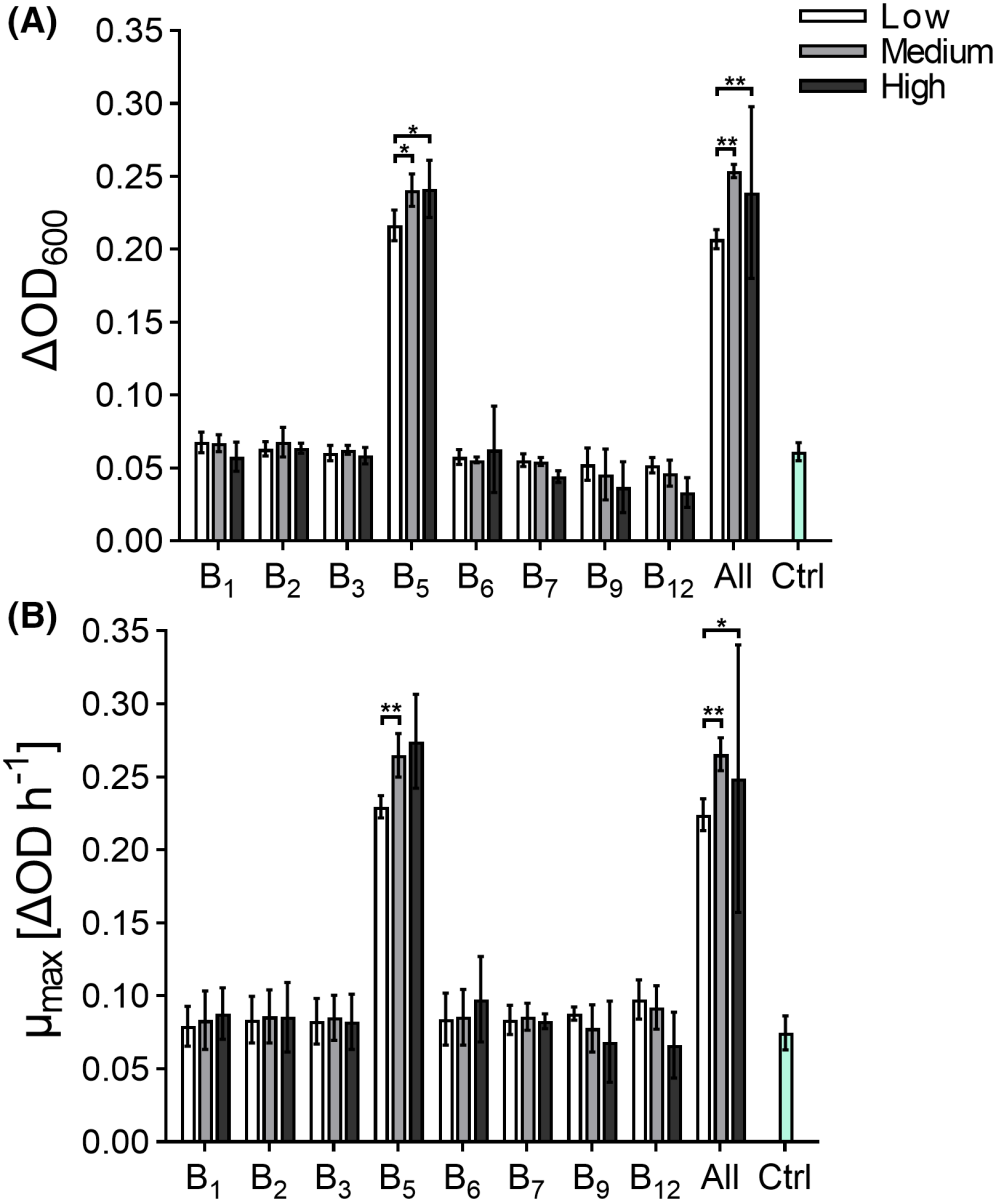
Impact of eight B‐vitamins at three different dosages on carrying capacity (ΔOD) and growth rate (ΔOD h^−1^) of *F. duncaniae* A2‐165 in microtitre plates. Bars represent the mean values of carrying capacity (ΔOD) (A) and μ_max_ (B) of quadruplicate growth experiments supplemented with either different B‐vitamins alone or all together (all) at low, medium, or high B‐vitamin concentration and compared to a control without vitamins (Ctrl). Statistics were performed by two‐way ANOVA following a post‐hoc test using Tukey HST, *p < 0.05, **p < 0.01.

Multiple B‐vitamin auxotrophies were reported for *F. duncaniae* A2‐165 in silico (Soto‐Martin et al., 2020). To investigate the effect of B‐vitamins on *F. duncaniae* A2‐165 growth, MM containing 10 g L^−1^ YP2 and 5 g L^−1^ glucose was supplemented with different essential B‐vitamins (B1, B2, B3, B5, B6, B7, B9 and B12) at three different dosages and tested alone or all combined and compared to a control without B‐vitamin addition. Growth of *F. duncaniae* A2‐165 was monitored in anaerobic incubations using 96‐well microtitre plates and compared to a control lacking B‐vitamin supplementation. Out of the eight tested B‐vitamins, only vitamin B5 induced growth promotion when supplied as a single component (Figure 4A,B). Supplementing a low dose of B5 (100 μg L^−1^) resulted in a significantly higher growth (ΔOD of 0.16 ± 0.08 and μ_max_ of 0.19 ± 0.06 ΔOD h^−1^) than for control condition without added B‐vitamins (0.06 ± 01 and 0.07 ± 01, *p* ≤ 0.01). Doubling the B5 dose further enhanced growth (ΔOD of 0.24 ± 0.01 and μ_max_ of 0.26 ± 0.02 ΔOD h^−1^, *p* = 0.016) but no further increase was observed with quadruplicated concentration (ΔOD of 0.23 ± 0.03 and μ_max_ of 0.25 ± 05 ΔOD h^−1^). Combining all eight B‐vitamins did not result in further additive growth effects compared to B5 supplementation alone, indicating that B5 was the only B‐vitamin responsible for growth promotion.

**FIGURE 5:**
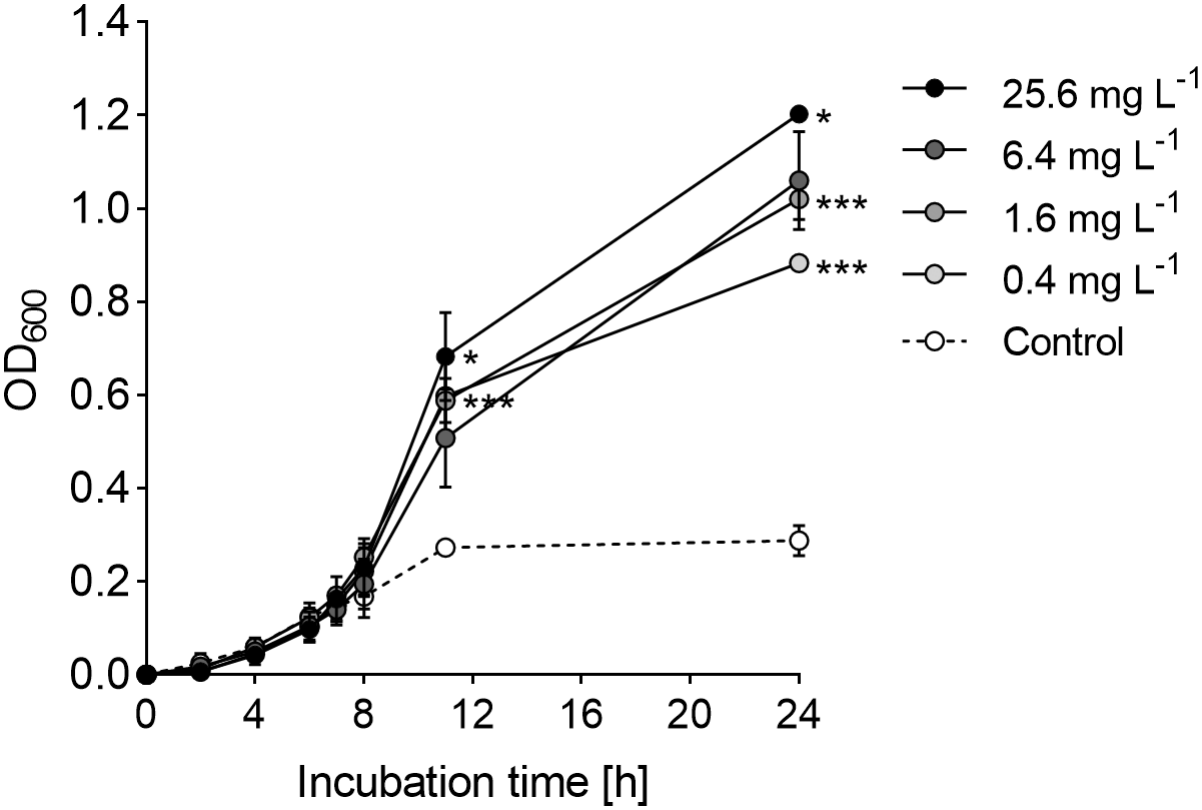
Growth kinetics of *F. duncaniae* A2‐165 upon B5 addition to MM medium at different dosages in Hungate tube fermentations. Dots represent the mean values of OD measurements of quadruplicate growth experiments in MM medium supplemented with different concentrations of vitamin B5 (0.4, 1.6, 6.4 and 25.6 mg L^−1^) and a control lacking B5. Statistics were performed by unpaired *t*‐test, comparing each concentration with the respective next higher concentration, *p < 0.05 and ***p < 0.001.

The small‐scale microtitre plate conditions may have led to restricted growth and limited the assessment of the full range of dose effects. To confirm the dose relationship of B5 and growth of *F. duncaniae* A2‐165, different B5 concentrations were added to MM in Hungate tube fermentations and growth was monitored by OD measurement over a 24‐h period. All tested concentrations of B5 showed a growth‐promoting effect on *F. duncaniae* A2‐165 (Figure 5). After 11 h of incubation, MM supplemented with different B5 concentrations had significantly higher ODs compared to the control without B5 (*p* < 0.001). B5‐supplemented conditions showed prolonged growth with a higher maximum OD recorded after 24 h of incubation compared to a maximal OD of 0.29 ± 0.03 after 11 h of incubation, followed by a plateau for the control. A significant effect of the B5 dose was shown, corresponding to a 3.1 and 4.2‐fold increase of the maximal OD for 0.4 mg L^−1^ and 25.6 mg L^−1^ B5, respectively, compared to the control (*p* < 0.001).

### Metabolic and growth response to B5 supplementation in an animal allergen‐free medium containing yeast extract

**FIGURE 6:**
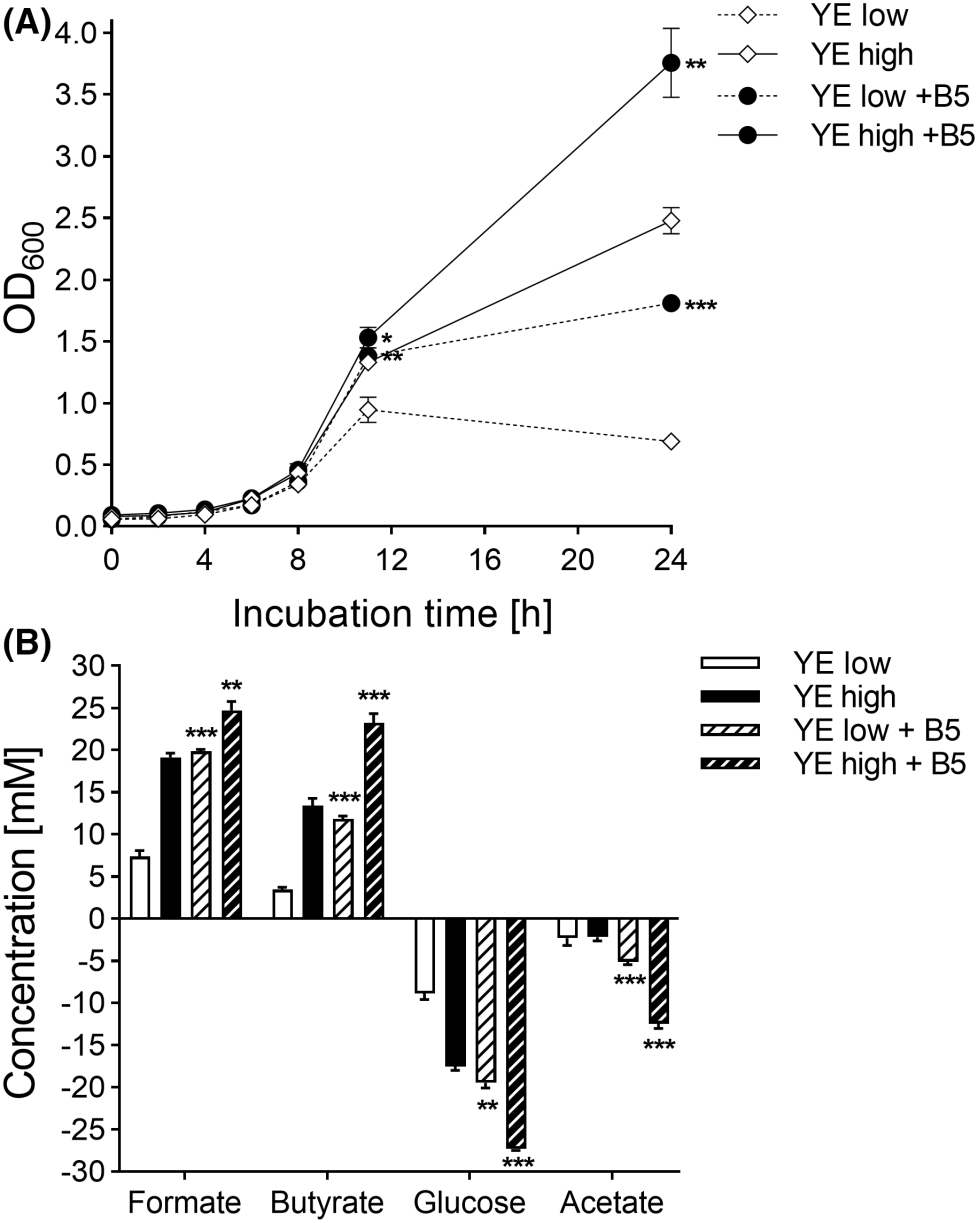
*F. duncaniae* A2‐165 cell yield, substrate consumption and metabolite production in Hungate tube fermentations using two different yeast extract (YE) dosages with or without B5 supplementation. Dots and bars represent the mean values of OD measurement (A) and end substrate and metabolite concentrations (B) of triplicate growth experiments in Hungate tubes containing MM supplemented with low (1.25 g L^−1^) and high YE2 doses (5.00 g L^−1^) and combined with and without vitamin B5 addition (6.4 mg L^−1^). Statistics were performed by unpaired *t*‐test, comparing for each YE concentration the OD values and metabolite concentrations with and without B5 addition, *p < 0.05, **p < 0.01 and ***p < 0.001.

To assess the impact of vitamin B5 on *F. duncaniae* A2‐165 growth in the presence of YE1, MM was supplemented with 6.4 mg L^−1^ B5 in combination with low (1.25 g L^−1^) and high concentrations (5.00 g L^−1^) of YE2. Growth of *F. duncaniae* A2‐165 was monitored over a 24‐h incubation in Hungate tubes, and results were compared to those obtained in MM lacking B5 but containing the corresponding dose of YE2. At the end of the incubation, substrate consumption and metabolite production were measured. To obtain an animal allergen‐free medium, casein peptone was replaced with YP2 that is produced using a fully allergen‐free process. Pre‐tests in Hungate tube and bioreactor fermentations confirmed that this substitution had no effect on *F. duncaniae* growth performance (Figures S1 and S2). The combination of vitamin B5 with YE resulted in significantly enhanced growth as compared to the condition without B5 (Figure 6A; *p* < 0.01). The effect was more pronounced at the low YE concentration, resulting in a 2.6‐fold increase in endpoint OD, from 0.7 ± 0.0 in the control to 1.8 ± 0.0 with addition of B5. A similar effect of B5 was observed at high YE condition, as a 1.5‐fold increase in OD was observed, from 2.5 ± 0.1 with YE alone to 3.8 ± 0.3 with B5 supplementation. Metabolite analysis confirmed the observed growth‐stimulating properties of B5, with increased substrate consumption and acetate and formate production with addition of B5 compared to YE alone (Figure 6B). Low YE concentration combined with B5 addition and high YE doses without B5 resulted in similar biomass and metabolite production.

**FIGURE 7:**
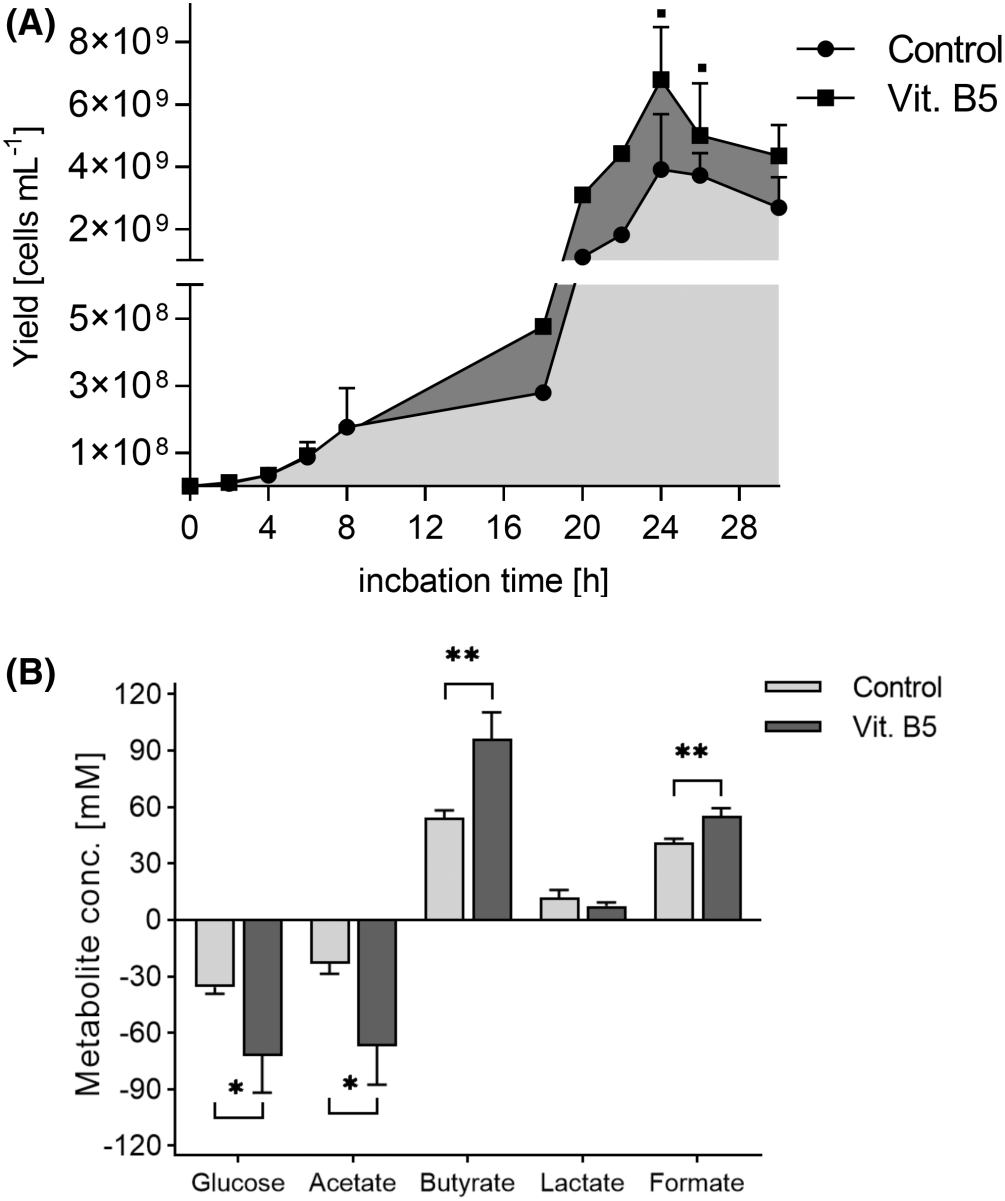
*F. duncaniae* A2‐165 cell yield, substrate consumption and metabolite production were enhanced under B5 supplementation in controlled bioreactor experiments. *F. duncaniae* A2‐165 was cultivated in controlled batch bioreactors in MM medium containing 40 g L^−1^ YE2, 10 g L^−1^ YP2 and 51.2 mg L^−1^ vitamin B5 and compared to a control without B5 addition. Cell yield (A) and metabolite concentrations (B) were measured in the spent media of each bioreactor over 30 h batch fermentation using quantitative real‐time polymerase chain reaction targeting the *F. duncaniae* A2‐165‐specific region of the 16S rRNA gene and HPLC‐RI, respectively. The values were normalized by six 16S rRNA gene copies to obtain the cell number per mL spent media. Dots represent average cell concentration at different time points of triplicated growth experiments, except for time point 18, 20 and 22 h that were only assessed for one replicate (A). Bars represent average values of end metabolite concentration (B) of triplicate growth experiments. Statistics were performed by unpaired *t*‐test, comparing the B5 group to controls, p < 0.01, *p < 0.05 and **p < 0.01.

The effect of B5 supplementation on *F. duncaniae* A2‐165 growth and metabolic activity was confirmed in bioreactor experiments using MM medium containing 25 g L^−1^ glucose (138 mM), 66 mM acetic acid, 40 g L^−1^ YE2 and 10 g L^−1^ YP2. The concentration of B5, previously used in Hungate tube fermentations, was increased by a factor of 8 to reach 54.2 mg L^−1^, corresponding to the same increase factor as that of YE1. Addition of B5 in the production medium resulted in a 1.5‐fold higher cell yield (6.8 ± 1.7 × 10^9^ cells mL^−1^) compared to the control condition with no B5 added (4.4 ± 1.0 × 10^9^ cells mL^−1^, *p* = 0.054; Figure 7A). B5 addition consistently led to a significant increase in substrate utilization (Figure 7B), resulting in a 77% increase of butyrate (96.5 ± 13.7 mM, *p* = 0.007) and a 33% higher formate production (55.4 ± 4.1 mM, *p* = 0.006) compared to the control reactors (54.5 ± 3.8 and 41.2 ± 2.1 mM, respectively).

## DISCUSSION

Several novel NGPs such as *F. duncaniae* have recently emerged and hold great promise as potential live biotherapeutic products to re‐establish homeostasis in the gastrointestinal tract (Martín & Langella, 2019). However, investigating and developing NGPs present substantial challenges, both for the scientific community and for industry since the candidates are generally difficult to cultivate at high cell concentrations owing to their complex nutritional requirements and high sensitivity to oxygen and other environmental stresses (De Filippis et al., 2022). Furthermore, NGPs should be produced in cGMP conditions for safe human use, namely, in animal allergen‐free culture media. In this study, we developed a culture medium containing a selected YE and supplemented with vitamin B5 that is free of animal‐derived ingredients, and yields high *F. duncaniae* A2‐165 cell concentrations in fully controlled bioreactors, of 7 × 10^9^ cells mL^−1^ after 24 h of incubation.

### Impact of YBN type on growth performance and metabolic activity

Starting with the high‐throughput growth assays, we observed a dose‐dependent growth‐promoting effect of the YEs in MM, while no such effect was recorded for YP or YCW. The absence of growth under YP and YCW conditions in MM medium indicates that specific growth factors necessary for *F. duncaniae* propagation are lacking, while YE likely supplies these components to a certain level. Compositional variations among different YBN types are the consequence of different production processes. As a general and basic rule, YE correspond to the water‐soluble fraction obtained from autolysed yeast cells, while YP represents the soluble fraction of yeast proteins hydrolysed by exogenous enzymes. Thus, YPs mainly serve as nitrogen sources in cultivation media. They are less rich in B‐vitamins (pantothenic acid, biotin and nicotinic acid) that are naturally present in YEs (Nancib et al., 1991). In addition, they usually have a wider peptide size distribution and lower level of free amino acids (Proust et al., 2019). Similarly, YCW is a good source of peptides and complex carbohydrates as its main components are mannoproteins and the two polysaccharides β‐glucan and chitin, but it lacks growth factors that are only present in the cytoplasm of yeast cells (Kollar et al., 1997; Stewart, 2017). Therefore, YPs and YCW are important sources of macronutrients but are limited in micronutrients essential for *F. duncaniae* propagation and should only be incorporated in a medium formulation that is supplemented with appropriate amounts of these essential growth factors or when combined with YE. As previously reported, the high‐throughput growth assay was a fast, simple and cost‐efficient method for kinetic measurements under anaerobic conditions (Bircher et al., 2018; Eini et al., 2013), which allowed the selection of YE1, YE2 and YE3 as the best performing YBN with the highest nutritional potential for further large‐scale production testing. A high *F. duncaniae* cell yield of ~5 × 10^9^ cells mL^−1^ was reached in controlled bioreactors by including 2%–4% YE1 (w/v) in the cultivation medium. To date, no published data is available on the cell yields of *Faecalibacterium* spp. produced under similar conditions or industrial‐like configurations. To the best of our knowledge, the only published data available is for a yield of 1 × 10^8^ cells mL^−1^ of *F. duncaniae* cultivated in Hungate tubes using a Brain Heart Infusion medium supplemented with 0.5% YE (Martín et al., 2017). Cell yields typically achieved in industrial probiotic production can range from 10^8^ to 10^11^ CFU mL^−1^, depending on factors such as the type of probiotic strain, fermentation conditions and medium used (Kumar et al., 2022). Surprisingly, above a certain amount added, the YE dosage had no significant effect on cell yields, which points towards a limiting growth factor(s) that cannot be compensated by a higher supplementation dose. Consistently, substrate utilization and metabolic yield are comparable between dosages but differ between YE type, with significantly higher butyrate production and substrate consumption in YE1 fermentations than with in fermentations with YE2 or YE3. Formation of butyrate over the acetyl coenzyme A (acetyl‐CoA) pathway requires net acetate consumption and relies on micronutrients such as thiamine (vitamin B1) and riboflavin (vitamin B2) (Soto‐Martin et al., 2020; Vital et al., 2017). Riboflavin plays a role in the electron transfer flavoprotein complex with butyryl‐CoA dehydrogenase, forming butyryl‐CoA from crotonyl‐CoA (Buckel & Thauer, 2018) while thiamine serves as cofactor of the pyruvate: ferredoxin 2‐oxidoreductase that forms acetyl‐CoA from pyruvate (Degnan et al., 2014). Both cofactors are naturally present in YE and may be key nutritional factors in directing *F. duncaniae* metabolism towards butyrate production. Since no additional vitamins were included in the cultivation medium, the supplied YEs are the only possible source of these nutritional elements. Thus, the high metabolic activity observed with YE1 might be an indication that it is a compositionally richer nutritional source for *F. duncaniae* production than the other two tested YEs. This finding highlights the importance to carefully screen and select the source of YE for optimal cell production.

### Effect of B‐vitamin supplementation on cell yield and metabolism

YCFA‐like medium promoted the growth of *F. duncaniae* in the high‐throughput growth assay, resulting in generally higher ΔOD than in MM, independent of the added YE. Interestingly, it also enabled *F. duncaniae* growth under YP2 conditions. This points towards nutritional limitations of YP2 that were partly compensated by the additional nutrients, including B‐vitamins supplied with the YCFA medium (Table S1). *Faecalibacterium* spp. were reported to be auxotrophic for vitamins B2, B3, B5, B6, B7 and B9 ‐ with a percentage of these vitamin biosynthesis genes varying between 17% and 75%—and prototrophic for vitamins B1, B7 and B12 (Rodionov et al., 2019; Soto‐Martin et al., 2020). However, the latter authors could demonstrate that including B_12_ in the cultivation medium had a growth promoting effect on *F. duncaniae* strain A2‐165 and different reports in literature suggest that this strain must benefits from the provision of other growth factors from microbiota community members (Fenn et al., 2017; Lebas et al., 2020; Sharma et al., 2019). When we tested the growth‐promoting effects of eight B‐vitamins in MM containing 1% YP2 (w/v), only pantothenate (vitamin B_5_) induced growth promotion, which is quite unexpected given the multiple B‐vitamin auxotrophies reported for that strain (Soto‐Martin et al., 2020) and by predictions based on published genome sequences of *F. duncaniae* strains annotated in the KEGG database. The combination of all eight B‐vitamins did not exhibit additional growth effects, suggesting that B5 was the primary nutritional factor responsible for promoting growth. This strong boosting effect might be explained by the high dependency of *F. duncaniae* on exogenous B_5_ for its growth and metabolism (Heinken et al., 2014). B5 is an essential precursor for the biosynthesis of coenzyme A (CoA), which is involved in a multitude of metabolic reactions and is a key factor for cell growth (Leonardi & Jackowski, 2007). Pantothenate is also a key coenzyme in sugar catabolism and fatty acid biosynthesis. Soto‐Martin et al. (2020) revealed in silico that *F. duncaniae* A2‐165 displays an incomplete pathway for CoA biosynthesis from pantothenate (75%). Previous metabolic studies and KEGG database consultation suggest that different precursors besides pantothenate, including S‐sulfonic acid‐type pantetheine‐related compounds, pantetheine and free CoA, can be utilized by *Faecalibacterium* for CoA biosynthesis. By adding 6.4 mg L^−1^ B_5_ to Hungate fermentations containing 5 g L^−1^ YE, we could enhance *F. duncaniae* butyrate production 2‐fold and growth 1.5‐fold. The measured OD of 3.8 ± 0.3 is to the best of our knowledge the highest OD reported for *F. duncaniae* culture in Hungate tubes (Lopez‐Siles et al., 2012; Martín et al., 2017). This confirms the importance of B5 for growth and metabolism of *F. duncaniae* when YE is supplied. Consequently, including B5 in the growth media formulation allows reduction in the amount of YE without compromising cell yield, which could possibly decrease medium costs.

### Additive effect of B5 on cell yield and metabolism in production tests

The positive effect of B_5_ addition on *F. duncaniae* growth and metabolism observed in Hungate tube fermentations was replicated in bioreactor experiments. Adding 51.2 mg L^−1^ B5 to the cultivation medium not only increased cell yield to 6.8 ± 1.7 × 10^9^ cells mL^−1^ and enhanced metabolite production, but also increased the butyrate:formate ratio. Acetate is required by *F. duncaniae* as a co‐substrate in one of the last steps in the butyrate production pathway where acetyl‐CoA is generated (Duncan, Barcenilla, et al., 2002; Duncan, Hold, et al., 2002). As vitamin B5 is a precursor of acetyl‐CoA, its supplementation might lead to more efficient acetate utilization and in return higher butyrate formation at the expense of formate production (Magnusdottir et al., 2015). Most of the available acetate (66 mM) was consumed by *F. duncaniae* under conditions containing B5 but not in the control lacking B5.

## CONCLUSION

The present study provides deeper insights into the growth performance of the NGP strain *F. duncaniae* A2‐165 upon supplementation with compositionally different YBNs. We could demonstrate that culture and metabolite production patterns of *F. duncaniae* A2‐165 are greatly influenced by the type of YBN provided, highlighting the importance of testing and selecting the optimal YBN ingredient for the commercial production of *F. duncaniae*. By replacing casein peptone with a combination of YE and YP, we were also able to define a full and efficient culture medium devoid of all allergenic ingredients of animal origin, as well as being compatible with cGMP production. Our current work emphasizes the importance of understanding specific nutritional requirements of the cultured strain so that the most suitable YBN and growth factors can be chosen to support biomass production at high cell density. In this respect, we identified vitamin B5 as a highly effective growth promoter for *F. duncaniae*. Fine tuning the culture medium composition by selecting or designing most suitable YBNs for a particular microbe and bioprocess could not only lead to increased cell yields while decreasing the manufacturing costs but might also support stability of the cultures during downstream processing and preservation.

## AUTHOR CONTRIBUTIONS

**Lea Bircher:** Conceptualization (equal); data curation (lead); formal analysis (lead); investigation (equal); methodology (lead); project administration (lead); supervision (lead); validation (lead); visualization (lead); writing – original draft (lead). **Alain M. Sourabié:** Conceptualization (equal); validation (equal); writing – review and editing (equal). **Marijana Paurevic:** Investigation (equal). **Janina Hochuli:** Investigation (equal). **Annelies Gerinaert:** Conceptualization (equal); writing – review and editing (equal). **Chloé Navas:** Resources (equal). **Benoît Drogue:** Resources (equal). **Christophe Lacroix:** Conceptualization (equal); funding acquisition (equal); validation (equal); writing – review and editing (equal).

## FUNDING INFORMATION

The work was funded by LeSaffre, 103 Rue Jean Jaurès, 94700 Maisons‐Alfors, France.

## CONFLICT OF INTEREST STATEMENT

CN, BD and AS were employed by the company Lesaffre (Procyles). The remaining authors declare that the research was conducted in the absence of any commercial or financial relationships that could be construed as a potential conflict of interest.

## Supporting information


Data S1:
