# Supplementary material for: Faecalibacterium duncaniae A2‐165 growth is strongly promoted by yeast extract and vitamin B5 in cGMP medium
Source: Microb Biotechnol. 2023 Nov 29;17(1):e14374. doi: 10.1111/1751-7915.14374 (PMC10832529; doi:10.1111/1751-7915.14374)
Supplement: Supplementary file 1 — Data S1: [file MBT2-17-e14374-s001.docx]

## Supplementary Figures

Supplementary Figure 1 Comparison of growth kinetics of F. duncaniae A2-165 in YCFA medium containing either casein or yeast peptone (YP2) in Hungate tube fermentations. Dots represent the mean values of OD measurements of triplicated growth experiments in YCFA medium supplemented with 2.5 g L^-1^ YE1 and 1 g L^-1^ casein peptone (*
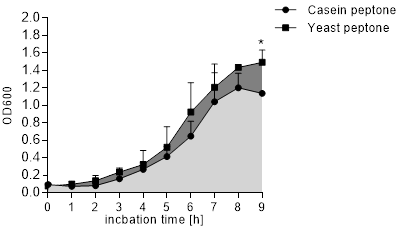
*) or YP2 (*
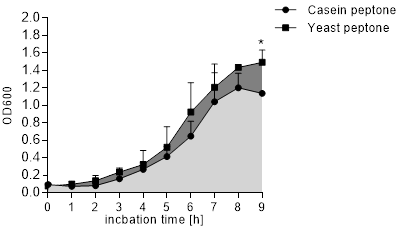
* ). Statistics were performed by unpaired t-test, comparing for each time point the condition containing casein peptone with the condition containing YP2 * p<0.05, ** p<0.01, *** p<0.001

Supplementary Figure 2 Comparison of growth kinetics of F. duncaniae A2-165 in MM medium containing either casein or yeast peptone (YP2) in bioreactor experiments. Dots represent F. duncaniae A2-165 cell concentration at different time points of single growth experiments in MM medium supplemented with 40 g L^-1^ YE1 and either 10 g L^-1^ casein peptone (*
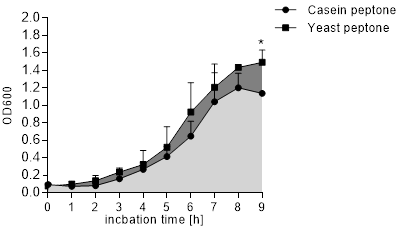
*) or YP2 (*
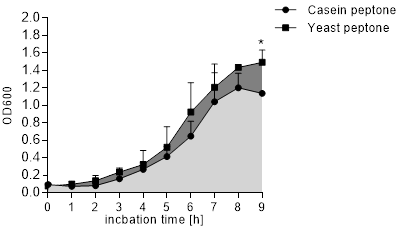
* ).

## Supplementary Tables

Supplementary Table 1 Composition of the minimal medium (MM)

| **Component*** | **g L^-1^** |
| --- | --- |
| Casein acid hydrolysate, from bovine milk | 2.00 |
| Sodium bicarbonate | 4.00 |
| Glucose | 5.00 |
| Potassium dihydrogen phosphate  Dipotassium hydrogen phosphate  Sodium chloride  Ammonium sulfate  Magnesium sulfate  Calcium chloride | 0.45  0.45  0.90  0.90  0.09  0.09 |
| Dipotassium hydrogen phosphate | 0.45 |
| Sodium chlorid | 0.90 |
| Ammonium sulfate | 0.90 |
| Magnesium sulfate | 0.09 |
| Calcium chloride | 0.09 |
| Acetic acid | 1.90 mL |
| Resazurin (1 mg mL^-1^) | 1.00 mL |
| L-cysteine hydrochloride monohydrate | 1.00 |

*All components were supplied by Sigma-Aldrich, Buchs, Switzerland, except potassium dihydrogen phosphate, dipotassium hydrogen phosphate and calcium cloride (VWR International Chemie GmbH, Dietikon Switzerland)

Supplementary Table 2 Composition of the Yeast-Casitone-Fatty-Acid-like medium (YCFA) without yeast extract

| **Component*** | **g L^-1^** |
| --- | --- |
| Casein acid hydrolysate, from bovine milk | 10.00 |
| Sodium bicarbonate | 4.00 |
| Glucose | 5.00 |
| Potassium dihydrogen phosphate  Dipotassium hydrogen phosphate  Sodium chloride  Ammonium sulfate  Magnesium sulfate  Calcium chloride | 0.45  0.45  0.90  0.90  0.09  0.09 |
| Dipotassium hydrogen phosphate | 0.45 |
| Sodium chlorid | 0.90 |
| Ammonium sulfate | 0.90 |
| Magnesium sulfate | 0.09 |
| Calcium chloride | 0.09 |
| Vitamin solution (10 mg L^-1^ biotin, 10 mg L^-1^ cobalamin, 30 mg L^-1^ p-aminobenzoic acid, 50 mg L^-1^ folic acid, 150 mg L^-1^ pyridoxamine) | 1.00 mL |
| Acetic acid | 1.9 mL |
| Hemin (0.5 mg mL^-1^) | 0.20 mL |
| Resazurin (1 mg mL^-1^) | 1.00 mL |
| L-cysteine hydrochloride monohydrate | 1.00 |

*All components were supplied by Sigma-Aldrich, Buchs, Switzerland, except potassium dihydrogen phosphate, dipotassium hydrogen phosphate and calcium cloride (VWR International Chemie GmbH, Dietikon Switzerland)

Supplementary Table 3 Experimental code and characteristics of the tested YBS. Six different yeast extracts and yeast peptones were selected for the high-throughput growth experiment.

| **Code** | **Characteristics** |
| --- | --- |
| YE1 | Yeast extract rich in nitrogen content, vitamins, and minerals |
| YE2 | Yeast extract enriched in nucleotides. Different batch for last production experiment (fully allergen-free production) |
| YE3 | Rich in different components from yeast cells and cell walls |
| YP1 | Yeast peptone with a high level of mid-size peptides (<5 res.) |
| YP2 | Yeast peptone with a medium level of mid-size and longer peptides (<10 res.) Produced using a fully allergen-free process |
| YCW | Enzymatically digest of yeast cell wall:  Rich in cell wall polysaccharides (β 1.3-1.6 glucan and mannans) |

Supplementary Table 4 Concentrations of B-vitamins tested for their growth-promoting potential on F. duncaniae A2-165

| Compound* | High dose | Medium dose | Low dose |
| --- | --- | --- | --- |
| Vitamin B1 | 200 µg L^-1^ | 100 µg L^-1^ | 50 µg L^-1^ |
| Vitamin B2 | 200 µg L^-1^ | 100 µg L^-1^ | 50 µg L^-1^ |
| Vitamin B3 | 200 µg L^-1^ | 100 µg L^-1^ | 50 µg L^-1^ |
| Vitamin B5 | 400 µg L^-1^ | 200 µg L^-1^ | 100 µg L^-1^ |
| Vitamin B7 | 400 µg L^-1^ | 200 µg L^-1^ | 100 µg L^-1^ |
| Vitamin B9 | 80 µg L^-1^ | 40 µg L^-1^ | 20 µg L^-1^ |
| Vitamin B12 | 20 µg L^-1^ | 10 µg L^-1^ | 5 µg L^-1^ |
| L-Arginine | 125 mg L^-1^ | 62.5 mg L^-1^ | 31.25 mg L^-1^ |
| L-Histidine | 150 mg L^-1^ | 75 mg L^-1^ | 37.5 mg L^-1^ |
| L-Leucine | 475 mg L^-1^ | 237.5 mg L^-1^ | 118.75 mg L^-1^ |
| L-Lysine | 437.5 mg L^-1^ | 218.75 mg L^-1^ | 109.375 mg L^-1^ |
| L-Phenylalanine | 750 mg L^-1^ | 375 mg L^-1^ | 187.5 mg L^-1^ |
| L-Tryptophan | 150 mg L^-1^ | 75 mg L^-1^ | 37.5 mg L^-1^ |

*All components were supplied by Sigma-Aldrich, Buchs, Switzerland

Supplementary Table 5 Composition of the production medium.

| Components* | Concentration [g L^-1^] |
| --- | --- |
| Yeast peptone | 10.00 |
| Yeast extract | 40.00 |
| Glucose | 25.00 |
| Acetic acid | 66 mM |
| K_2_HPO_4_ | 0.15 |
| KH_2_PO_4_ | 0.15 |
| NaCl | 0.9 |
| (NH_4_)_2_SO_4_ | 0.9 |
| MgSO_4_ | 0.09 |
| CaCl_2_ | 0.09 |
| NaHCO_3_ | 4.0 |
| L-cysteine HCl | 1.00 |

*All components were supplied by Sigma-Aldrich, Buchs, Switzerland, except potassium dihydrogen phosphate, dipotassium hydrogen phosphate and calcium cloride (VWR International Chemie GmbH, Dietikon Switzerland)

**Supplementary Table 6** **Composition of qPCR reaction mix per sample**

| **Component** | **Volume per reaction [µL]** |  | **Supplier** |
| --- | --- | --- | --- |
| SensiFAST^TM^ SYBR® No-ROX mastermix | 5 |  | Labgene Scientific, Châtel-Saint-Denis, Switzerland |
| MiliQ water | 3 |  |  |
| Forward primer (10 µM) | 0.5 |  | Microsynth, Balgach, Schwitzerland |
| Backward primer (10 µM) | 0.5 |  | Microsynth, Balgach, Schwitzerland |
| DNA template | 1 |  |  |
| Total volume | 10 |  |  |

Supplementary Table 7 Tukey multiple comparison of means of the carrying capacity and max. growth rate from the high-throughput screening of six different YBNs and three dosages.

| **Factor** | **Comparison** | **ΔOD** | | | | | **μ_max_** | | | | |
| --- | --- | --- | --- | --- | --- | --- | --- | --- | --- | --- | --- |
|  |  | **Δ in means** | **95% CI for mean** | | **p adj** |  | **Δ in means** | **95% CI for mean** | | **p adj** |  |
|  |  |  | **lower** | **upper** |  |  |  | **lower** | **upper** |  |  |
| Product | YCW-Ctrl | 0.0 | 0.0 | 0.1 | 0.918 |  | 0.0 | -0.1 | 0.1 | 0.992 |  |
|  | YE1-Ctrl | 0.6 | 0.5 | 0.7 | 0.000 | *** | 0.7 | 0.6 | 0.8 | 0.000 | *** |
|  | YE2-Ctrl | 0.6 | 0.5 | 0.6 | 0.000 | *** | 0.7 | 0.6 | 0.8 | 0.000 | *** |
|  | YE3-Ctrl | 0.5 | 0.4 | 0.5 | 0.000 | *** | 0.5 | 0.4 | 0.6 | 0.000 | *** |
|  | YP1-Ctrl | 0.0 | -0.1 | 0.0 | 0.742 |  | 0.5 | 0.3 | 0.6 | 0.000 | *** |
|  | YP2-Ctrl | 0.1 | 0.0 | 0.1 | 0.169 |  | 0.0 | -0.1 | 0.2 | 0.848 |  |
|  | YE1-YCW | 0.6 | 0.5 | 0.6 | 0.000 | *** | 0.7 | 0.6 | 0.769 | 0.000 | *** |
|  | YE2-YCW | 0.5 | 0.5 | 0.6 | 0.000 | *** | 0.7 | 0.6 | 0.753 | 0.000 | *** |
|  | YE3-YCW | 0.4 | 0.4 | 0.5 | 0.000 | *** | 0.5 | 0.4 | 0.553 | 0.000 | *** |
|  | YP1-YCW | -0.1 | -0.1 | 0.0 | 0.007 |  | 0.4 | 0.3 | 0.506 | 0.000 | *** |
|  | YP2-YCW | 0.0 | 0.0 | 0.1 | 0.501 |  | 0.0 | -0.1 | 0.097 | 0.979 |  |
|  | YE2-YE1 | 0.0 | -0.1 | 0.0 | 0.483 |  | 0.0 | -0.1 | 0.066 | 0.999 |  |
|  | YE3-YE1 | -0.1 | -0.2 | -0.1 | 0.000 | *** | -0.2 | -0.3 | -0.133 | 0.000 | *** |
|  | YP1-YE1 | -0.6 | -0.7 | -0.6 | 0.000 | *** | -0.3 | -0.3 | -0.181 | 0.000 | *** |
|  | YP2-YE1 | -0.5 | -0.6 | -0.5 | 0.000 | *** | -0.7 | -0.7 | -0.589 | 0.000 | *** |
|  | YE3-YE2 | -0.1 | -0.2 | -0.1 | 0.000 | *** | -0.2 | -0.3 | -0.124 | 0.000 | *** |
|  | YP1-YE2 | -0.6 | -0.7 | -0.6 | 0.000 | *** | -0.2 | -0.3 | -0.171 | 0.000 | *** |
|  | YP2-YE2 | -0.5 | -0.6 | -0.5 | 0.000 | *** | -0.7 | -0.7 | -0.580 | 0.000 | *** |
|  | YP1-YE3 | -0.5 | -0.5 | -0.4 | 0.000 | *** | -0.1 | -0.1 | 0.028 | 0.463 |  |
|  | YP2-YE3 | -0.4 | -0.5 | -0.3 | 0.000 | *** | -0.5 | -0.5 | -0.381 | 0.000 | *** |
|  | YP2-YP1 | 0.1 | 0.0 | 0.1 | 0.000 | *** | -0.4 | -0.5 | -0.327 | 0.000 | *** |
| Dose | Low-High | -0.3 | -0.3 | -0.3 | 0.000 | *** | -0.4 | -0.4 | -0.3 | 0.000 | *** |
|  | Medium-High | -0.2 | -0.2 | -0.1 | 0.000 | *** | -0.2 | -0.2 | -0.1 | 0.000 | *** |
|  | Medium-Low | 0.1 | 0.1 | 0.2 | 0.000 | *** | 0.2 | 0.1 | 0.2 | 0.000 | *** |

**Supplementary Table 8 Two-way ANOVA with interaction term of the carrying capacity and max. growth rate from the high-throughput screening of six different YBNs and three dosages.**

| **Medium** | **Growth parameter** | **Factor** | **Degree of freedom** | **Sum of squares** | **Mean of squares** | **F-value** | **Pr(>F)** |  |
| --- | --- | --- | --- | --- | --- | --- | --- | --- |
| MM | ΔOD | Product | 6 | 10.9 | 1.8 | 513.3 | 0.000 | *** |
|  |  | Dose | 2 | 2.0 | 1.0 | 276.5 | 0.000 | *** |
|  |  | Product:Dose | 10 | 1.7 | 0.2 | 46.6 | 0.000 | *** |
|  |  | Residuals | 128 | 0.5 | 0.0 |  |  |  |
|  | μ_max_ | Product | 2 | 22.9 | 3.8 | 0.2 | 0.000 | *** |
|  |  | Dose | 1 | 0.0 | 0.0 | 0.4 | 0.825 |  |
|  |  | Product:Dose | 10 | 0.2 | 0.0 |  | 0.963 |  |
|  |  | Residuals | 127 | 8.6 | 0.1 |  |  |  |
| YCFA | ΔOD | Product | 6 | 12.0 | 2.0 | 265.3 | 0.000 | *** |
|  |  | Dose | 2 | 2.9 | 1.4 | 191.7 | 0.000 | *** |
|  |  | Product:Dose | 10 | 1.6 | 0.2 | 21.7 | 0.000 | *** |
|  |  | Residuals | 124 | 0.9 | 0.0 |  |  |  |
|  | μ_max_ | Product | 6 | 22.4 | 3.7 | 84.5 | 0.000 | *** |
|  |  | Dose | 2 | 0.3 | 0.1 | 3.3 | 0.041 | * |
|  |  | Product:Dose | 10 | 1.4 | 0.1 | 3.2 | 0.001 | *** |
|  |  | Residuals | 128 | 5.7 | 0.0 |  |  |  |

Supplementary Table 9 Tukey multiple comparison of means of the metabolite and yield data of the production trial with three different YBNs and two dosages.

|  | **Factor** | **Comparison** | **Δ in means** | **95% CI for mean** | | **p adj** |  |
| --- | --- | --- | --- | --- | --- | --- | --- |
|  |  |  |  | **lower** | **upper** |  |  |
| Butyrate [mM] | Product | YE2-YE1 | -30.1 | -44.2 | -16.0 | 0.000 | *** |
|  |  | YE3-YE1 | -23.4 | -38.2 | -8.7 | 0.003 | ** |
|  |  | YE3-YE2 | 6.7 | -8.1 | 21.4 | 0.477 |  |
|  | Dose | 4%-2% | 7.4 | -2.3 | 17.1 | 0.124 |  |
| Lactate [mM] | Product | YE2-YE1 | 2.4 | -4.8 | 9.7 | 0.662 |  |
|  |  | YE3-YE1 | 6.8 | -0.8 | 14.4 | 0.080 |  |
|  |  | YE3-YE2 | 4.4 | -3.2 | 12.0 | 0.306 |  |
|  | Dose | 4%-2% | 0.0 | -5.0 | 5.0 | 0.998 |  |
| Formate [mM] | Product | YE2-YE1 | -9.7 | -27.1 | 7.7 | 0.337 |  |
|  |  | YE3-YE1 | -21.1 | -39.3 | -2.8 | 0.024 | * |
|  |  | YE3-YE2 | -11.4 | -29.6 | 6.8 | 0.261 |  |
|  | Dose | 4%-2% | -10.2 | -22.1 | 1.8 | 0.090 |  |
| Acetate [mM] | Product | YE2-YE1 | 24.3 | 8.4 | 40.2 | 0.004 |  |
|  |  | YE3-YE1 | 12.6 | -4.1 | 29.3 | 0.152 |  |
|  |  | YE3-YE2 | -11.7 | -28.4 | 5.0 | 0.192 |  |
|  | Dose | 4%-2% | -11.1 | -22.0 | -0.1 | 0.048 | * |
| Glucose [mM] | Product | YE2-YE1 | 26.0 | 10.7 | 41.3 | 0.002 | ** |
|  |  | YE3-YE1 | 21.1 | 5.1 | 37.2 | 0.011 | * |
|  |  | YE3-YE2 | -4.9 | -20.9 | 11.2 | 0.711 |  |
|  | Dose | 4%-2% | 1.1 | -9.5 | 11.7 | 0.826 |  |
| Yield [cells mL^-1^] | Product | YE2-YE1 | 5.6E+08 | -3.7E+09 | 4.8E+09 | 0.936 |  |
|  |  | YE3-YE1 | -2.8E+09 | -7.3E+09 | 1.6E+09 | 0.254 |  |
|  |  | YE3-YE2 | -3.4E+09 | -7.8E+09 | 1.1E+09 | 0.151 |  |
|  | Dose | 4%-2% | 1.4E+09 | -1.5E+09 | 4.3E+09 | 0.315 |  |

Signif. codes: 0 ‘***’ 0.001 ‘**’ 0.01 ‘*’ 0.05 ‘.’ 0.1 ‘ ’ 1

Supplementary Table 10 Two-way ANOVA without interaction term of the metabolite and yield date from the production trial with three different YBNs and two dosages.

|  | **Factor** | **Degree of freedom** | **Sum of squares** | **Mean of squares** | **F-value** | **Pr(>F)** |  |
| --- | --- | --- | --- | --- | --- | --- | --- |
| Butyrate [mM] | Product | 2 | 2965.5 | 1482.7 | 17.4 | 0.000 | *** |
|  | Dose | 1 | 232.2 | 232.2 | 2.7 | 0.122 |  |
|  | Residuals | 13 | 1105.3 | 85.0 |  |  |  |
| Lactate [mM] | Product | 2 | 129.4 | 64.7 | 2.9 | 0.093 | . |
|  | Dose | 1 | 0.0 | 0.0 | 0.0 | 0.998 |  |
|  | Residuals | 13 | 293.5 | 22.6 |  |  |  |
| Formate [mM] | Product | 2 | 1209.3 | 604.7 | 4.7 | 0.030 | * |
|  | Dose | 1 | 440.6 | 440.6 | 3.4 | 0.089 | . |
|  | Residuals | 13 | 1689.9 | 130.0 |  |  |  |
| Acetate [mM] | Product | 2 | 1772.2 | 886.1 | 8.1 | 0.005 | ** |
|  | Dose | 1 | 523.3 | 523.3 | 4.8 | 0.047 | * |
|  | Residuals | 13 | 1414.9 | 108.8 |  |  |  |
| Glucose [mM] | Product | 2 | 2262.1 | 1131.0 | 11.2 | 0.001 | ** |
|  | Dose | 1 | 5.1 | 5.1 | 0.1 | 0.825 |  |
|  | Residuals | 13 | 1314.0 | 101.1 |  |  |  |
| Yield [cells mL^-1^] | Product | 2 | 3.5E+19 | 1.7E+19 | 2.237 | 0.146 |  |
|  | Dose | 1 | 8.5E+18 | 8.5E+18 | 1.103 | 0.313 |  |
|  | Residuals | 13 | 1.0E+20 | 7.7E+18 |  |  |  |

Signif. codes: 0 ‘***’ 0.001 ‘**’ 0.01 ‘*’ 0.05 ‘.’ 0.1 ‘ ’ 1
